# Fuse binding protein antagonizes the transcription activity of tumor suppressor protein p53

**DOI:** 10.1186/1471-2407-14-925

**Published:** 2014-12-08

**Authors:** Updesh Dixit, Zhihe Liu, Ashutosh K Pandey, Ramesh Kothari, Virendra N Pandey

**Affiliations:** Department of Microbiology, Biochemistry and Molecular Genetics, New Jersey Medical School, Rutgers Biomedical Health Sciences, Rutgers University, 185 South Orange Avenue, Newark, NJ 07103 USA; Guangzhou Institute of Traumatic Surgery, Guangzhou Red Cross Hospital, Medical College, Jinan University, Guangzhou, 510220 China

**Keywords:** FUSE binding protein, Hepatocellular carcinoma, Tumor suppressor protein p53

## Abstract

**Background:**

FUSE binding protein1 (FBP1) is a transactivator of transcription of human *c-myc* proto-oncogene and expressed mainly in undifferentiated cells. It is also present in differentiated normal cells albeit with very low background. FBP1 is abundantly expressed in the majority of hepatocellular carcinoma tumors and has been implicated in tumor development. Although it down-regulates the expression of proapoptotic p21 protein, it is not known whether FBP1 also interacts and antagonizes the function of tumor suppressor protein p53.

**Methods:**

Western blotting was carried out to detect the expression level of FBP1, p21 and p53, and also p53 regulatory factors, BCCIP and TCTP; real-time quantitative PCR was done to determine the fold change in mRNA levels of target proteins; immunoprecipitation was carried out to determine the interaction of FBP1 with p53, BCCIP and TCTP. Cells stably knockdown for either FBP1; p53 or BCCIP were examined for p53 reporter activity under normal and radiation-induced stress.

**Results:**

FBP1 physically interacted with p53, impairing its transcription activity and reducing p53-mediated sensitivity to cellular stress. Knockdown of FBP1 expression activated p53-mediated response to cellular stress while transient expression of FBP1 in FBP-knockdown cells restored the inhibition of p53 activity. FBP1 not only interacted with both BCCIP and TCTP, which, respectively, function as positive and negative regulators of p53, but also regulated their expression under cellular stress. In FBP knockdown cells, TCTP expression was down-regulated under radiation-induced stress whereas expression of BCCIP and p21 were significantly up-regulated suggesting FBP1 as a potential regulator of these proteins. We hypothesize that the FBP1-mediated suppression of p53 activity may occur via preventing the interaction of p53 with BCCIP as well as by FBP1-mediated regulation of p53 regulatory proteins, TCTP and BCCIP. Since FBP1 suppresses p53 activity and is overexpressed in most HCC tumors, it may have a possible role in tumorigenesis.

**Conclusion:**

FBP1 physically interacts with p53, functions as a regulator of p53-regulatory proteins (TCTP and BCCIP), and suppresses p53 transactivation activity under radiation-induced cellular stress. Since it is abundantly expressed in most HCC tumors, it may have implication in tumorigenesis and thus may be a possible target for drug development.

## Background

The tumor suppressor protein, p53, is a multifunctional factor that plays key roles in cell growth and death [1]. When activated by various cellular stresses that disrupt the fidelity of DNA replication and cell division, such as radiation-induced DNA damage, p53 induces transcriptional activation of specific target genes, including cell-cycle regulatory and proapoptotic factors that regulate cell fate. After DNA damage in a cell, the p53 pathway produces a set of proteins that are directly involved in DNA repair processes [2].

Loss of function of p53 as a consequence of mutation has been shown in 50% of human cancers [3]. Animal models lacking p53 have a higher predisposition to cancer [4, 5]. The ability of p53 to eliminate cells that potentially may become cancerous relies on its ability to induce programmed cell death, by regulating the expression of apoptotic genes such as PUMA [6]. In contrast, 50% of human cancers harbor transcriptionally active p53 in which the tumor suppression activity may have been antagonized by other mechanisms. For example, overexpression of MDM2 promotes degradation of p53 [7] while overexpression of TCTP (fortilin) blocks the binding of p53 with Bax and inhibits p53-dependent apoptosis [8]. Some proteins can promote p53 transcription activity; these include, BCCIP, a BRCA2 and CDKN1A (p21, Cip1, and Waf1) that is important in DNA repair [9]. Abrogation of the interaction between p53 and TCTP-like protein can retrieve p53 tumor-inhibiting activity. Small molecules, such as nutlins [10, 11] and RITA [12] can retrieve p53 tumor-suppressing activity by inhibiting the interaction between MDM2 and p53 or binding with p53. The mechanisms involved in impairing p53 transcription activity are potentially important for drug development for cancer treatment.

The p53 in Huh7 cells and cell lines derived from it carries a mutation at codon 220 (Y220C) which has been shown to be transcriptionally inactive [13–15]. In this report, we demonstrated that mutant p53 in Huh7 cells is transcriptionally active but remained suppressed due to its interaction with fuse binding protein1 (FBP1). FBP1 regulates the transcription of the *c-myc* gene by binding to an element called the far-upstream element (FUSE) [16–18]. FBP1, which is over-expressed in 80% of HCC [19], physically interact with p53 and suppresses its transcription activity. While FBP-knockdown (FBP-kd) significantly activated p53 transcription activity, transient expression of FBP1 in FBP-kd Huh7 cells restored the control phenotype inhibiting p53 functions suggesting a novel mechanism by which p53 is impaired in this cell line with the implication in the development of HCC tumor.

## Methods

### Cell culture

Huh7 liver cancer cells were grown in Dulbecco’s modified Eagle medium (DMEM) from Sigma (Saint Louis, MO) supplemented with 10% fetal bovine serum (Hyclone, Logan, UT), 100 units/ml of nonessential amino acids (Sigma), and 100 μg/ml of penicillin and streptomycin sulfate (Sigma). Cells were grown at 37°C with 5% CO_2_.

### Construction of stably transformed Huh7 cells knocked down for FBP1, p53 or BCCIP

We generated stably transduced Huh7 cells with lentivirus vectors encoding shRNA targeting FBP1, p53, or BCCIP (Santa Cruz, CA) or with empty vector alone (SC-108080) following the manufacturer’s protocol. As a control, Huh7 cells were infected with control lentiviral particles with empty vector (Santa Cruz, SC-108080). Stable clones were selected after several passages via puromycin selection in DMEM medium containing three μg/mL of puromycin (Santa Cruz, CA).Stable knockdown of expression of targeted protein was confirmed by Western blot analysis as compared to cells transformed with a vector alone.

### Transient expression of FBP1 in FBP-kd cells

**Figure 1:**
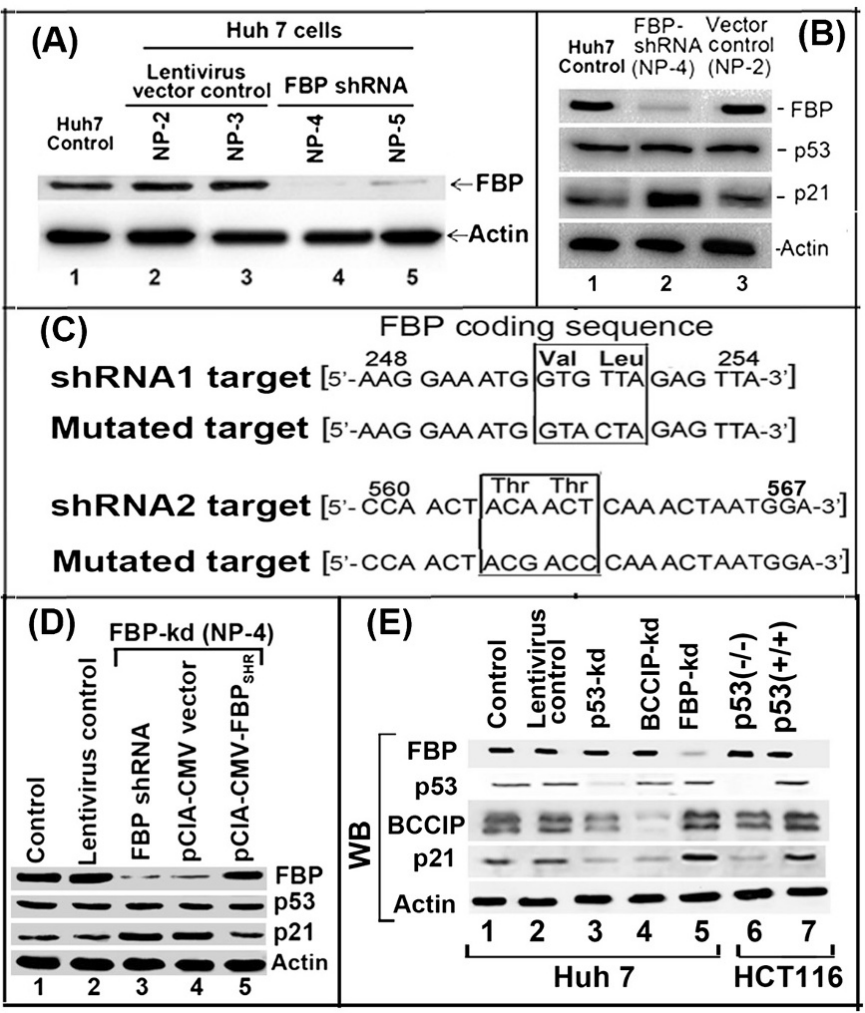
**FBP negatively affects p21 and BCCIP expression in Huh7 cells. (A)** Stably FBP-knockdown (FBP-kd) Huh7 cells by expressing FBP1 targeting shRNA: Huh7 cells were transfected with lentivirus vector encoding FBP1 targeting shRNAs or with empty vector alone. Stable clones (NP-4, NP-5) were selected after several passages via puromycin selection and were confirmed for stable knockdown of FBP1 expression by Western blot analysis as compared to cells transformed with the vector alone (NP-2, NP-3). **(B)**. Expression of p53 and p21 in FBP-kd cells. Huh7 cells stably transduced with FBP1 shRNA (NP-4) or empty vector alone (NP-2) were grown for 48 h. Cell lysates were normalized with respect to protein and Western blotted for the expression of FBP1, p53, p21, and actin. Lane 1, control Huh7 cells; lane 2, FBP-kd Huh7 cells; lane 3, Huh7 cells transduced with a lentivirus vector only. **(C)** Construction of FBP1 expression clone resistant to FBP1-shRNA. The FBP1-shRNA targeted sequences spanning codons 248 to 254 and from 560 to 567 were subjected to point mutation in FBP1 expression clone (pCIA-CMV-FBP) without changing the amino acid sequence. **(D)** Transient expression of FBP1 in FBP-kd cells suppressed the expression of p21. FBP-kd cells were transfected with shRNA resistant FBP1 expression clone ((pCIA-CMV-FBP_SHR_) or empty pCIA-CMV vector; 48-h later cells lysates were Western blotted for FBP1, p53 and p21 and Actin. Lane 1, control Huh7 cells, lane 2, Huh7 cells transduced with lentiviral vector alone; lane 3, FBP-kd cells; lane 4, FBP-kd cells transfected with empty vector; lane 5, FBP-kd cells transfected with shRNA resistant FBP1 expression clone. **(E)** Expression level of p21 in control and p53-kd, BCCIP-kd, and FBP-kd Huh7 cells. Lane1, control Huh7 cells; lane 2, lentivirus vector control; lane 3, p53-kd; lane 4, BCCIP-kd and lane 5, FBP-kd Huh7 cells. A colon cancer cell line, HCT116 without p53 (-/-) (lane 6) or with wild-type p53 (+/+) (lane 7) was used as controls.

For transient expression of FBP in FBP-kd cells, we constructed shRNA resistant FBP expression clone (pCIA-CMV-FBP) by mutating the siRNA target sequence without changing the amino acid sequence. The lentivirus based FBP1 shRNA targeting codons 248–254 in the central domain and codon 560–566 in the C-terminal region of FBP1. Point mutations were carried out at codons 251, 252, 562 and 563 without any change in the amino acid sequence (Figure 1C). We confirmed the shRNA resistance by transient expression of FBP1 in FBP-kd cells.

### Expression and purification of p53

*E. coli* BL21 cells transformed with pET11GTK-p53 plasmid were grown at 37°C in LB medium with 100 μg/ml ampicillin until an OD_595_ of 0.5 was achieved; the cells were then supplemented with 0.4 mM IPTG. Cells were incubated at 25°C for 3 h with vigorous shaking, then chilled and harvested by centrifugation. The cell pellets were washed once with 50 mM Tris–HCl containing 0.15 M NaCl, then resuspended in lysis buffer containing 0.1 M KCl, 2 mM EDTA, 20% glycerol, 25 mM HEPES buffer (pH7.6), 2 mM DTT, and 1× cocktail of protease inhibitors (R1321, Fermentas). We treated the cells with lysozyme (100 μg/ml) containing 0.1% NP40. After 15 min incubation at 4°C, cells were subjected to three cycles of freezing at -80°C and thawing, then sonicated at amplitude 40 with three 15-sec pulses. The lysed cells were centrifuged at 14,000 g at 4°C. The supernatant was mixed with glutathione-Sepharose beads (Pharmacia) at a ratio of 0.5 mg beads per ml of supernatant. The suspension was incubated at 4°C for 1 h and centrifuged at 1,500 rpm for 1 min. Beads were placed in a small column and washed extensively with wash buffer containing 50 mM Tris–HCl (pH 8.0) and 5% glycerol. The washed beads, resuspended in 0.5 ml of 10 mM reduced glutathione (Sigma-Aldrich), were incubated for 15 min at room temperature, then centrifuged. The supernatant fraction containing eluted GST-tagged p53 were pooled and diluted to 15-fold with a buffer containing 20 mM HEPES (pH7.5), 50 mM NaCl, 0.1 mM EDTA, 10 mM β-mercaptoethanol, 10% glycerol and protease cocktail inhibitor (Roche, USA). The diluted fraction was then applied onto the FPLC Hi-Trap heparin column (Pharmacia) and washed extensively with the same buffer. We eluted p53 protein with a linear gradient (0% to 80%) of 1.5 M NaCl in the same buffer for 20 min (1 ml/min). Eluted fractions showing greater than 95% purity on SDS-PAGE (8%) were dialyzed against 50 mM sodium phosphate (pH 7.8) containing 100 mM NaCl.

### Expression and purification of FBP1

We transformed *E. coli* Rosetta (DE3) cells with pET28a-FBP1 encoding His-tagged FBP1. Cells were grown at 37°C in LB medium containing 30 μg/ml of kanamycin until an OD_595_ 0.4 was achieved. The culture medium was cooled to 18°C and supplemented with 0.5 mM IPTG. After 16 h of incubation with vigorous shaking at 18°C, cells were harvested and resuspended in a lysis buffer [20 mM Tris–HCl pH 7.4, 200 mM NaCl, 1 mM β-mercaptoethanol, 10% glycerol, 1% triton-X 100, 5 mM imidazole]. The suspension was supplemented with 2 mg/ml of ProteoBlock protease inhibitor cocktail (Fermentas) and 2 mg/ml of lysozyme. After 15 min incubation on ice, cells were sonicated and centrifuged at 14,000 *g* for 30 min. The clear supernatant was applied onto the Ni-NTA column preequilibrated with binding buffer [20 mM Tris–HCl pH 7.4, 200 mM NaCl, 10% glycerol, and 5 mM imidazole]. The column was washed with the same buffer successively containing 30 mM and 50 mM of imidazole. His tagged FBP1 was eluted from the column with 200 mM imidazole in the same buffer. The eluted fractions were pooled, diluted 2-fold with a buffer containing 20 mM Tris–HCl pH 7.5, 5% glycerol, 0.5% NP-40, and 1 mM β-mercaptoethanol) and applied onto the Hi-Trap Heparin column (Pharmacia). After washing the column extensively with the same buffer, FBP1 was eluted with a linear gradient (0% to 80%) of 1 M KCl in the same buffer for 20 min (1 ml/min). Eluted fractions showing more than 95% purity on SDS-PAGE (8%) were pooled and dialyzed against buffer containing 50 mM Tris–HCl (pH 7.5), 2 mM DTT, 100 mM NaCl, and 50% glycerol.

### Expression and purification of BCCIP

We transformed *E. coli* Rosetta (DE3) cells separately with pET28a-BCCIPα and pET28a-BCCIP β encoding His-tagged proteins. The transformed cells were grown at 37°C in LB medium containing 30 μg/ml of kanamycin until an OD_595_ 0.4 was achieved. The medium was cooled to 26°C and then supplemented with IPTG to a final at 1.0 mM concentration. Cells were further incubated at 26°C for 16 h with vigorous shaking, then chilled and harvested by centrifugation. The cell pellets were resuspended in a lysis buffer supplemented with protease inhibitor cocktail and lysozyme as described. The cell lysates were sonicated; centrifuged and clear supernatant was applied onto Ni-NTA column and eluted as described above for FBP1 purification. Fractions of 0.5 mL were collected and analyzed by SDS-PAGE. The peak fraction with greater than 95% purity was dialyzed and stored in -20°C.

### Preparation of cell extract and western blotting

We washed the Huh7 cells with PBS three times, then lysed them at room temperature in lysis buffer containing 1% SDS, 10% glycerol, 1 mM DTT and 10 mM Tris–HCl, pH 6.8. We boiled the cell lysates for 5 min, cooled at room temperature, then centrifuged at 13,000 rpm for 10 min at 4°C. We saved the supernatant and determined the protein concentration (DC protein assay, Bio-Rad) in each sample. An aliquot of the supernatant equivalent to 15 μg protein was resolved on 8% SDS-PAGE, and then transferred to a nitrocellulose membrane (Schleicher and Schuell Bioscience). The membrane was first blocked by 5% non-fat dry milk in PBS buffer containing 0.5% Tween 20 for 1 h at room temperature. The membrane was then washed 4–5 times with the same buffer and treated overnight at 4°C with 1:1000 diluted primary antibody of the selected protein target (Santa Cruz Biotechnology). The membrane was washed again with the same buffer four times, then treated with 1:5,000 dilution of horseradish-peroxidase-conjugated secondary antibody for 1 h at room temperature and washed thoroughly. The membrane was then incubated with HRP substrate (Western Lightning Chemiluminescence Reagent, PerkinElmer Life Sciences) for 10 sec to 1 min, exposed on FluorChem M or Kodak X-Omat AR x-ray film (Eastman Kodak).

### Radiation survival assay

We determined the radiation sensitivity of Huh7 cells (FBP1 normal and knockdown) by colony formation experiments. We first did a pilot experiment with control Huh7 cells to determine the number of cells to be plated to obtain 50–150 surviving colonies per dish after a γ-irradiation dose of 3Gy. Cells were plated on 100-mm dishes 24 h before radiation treatment. We used a Cs-137 γ- irradiator to deliver 3-Gy to cells, and then incubated the irradiated cells for 14 days. The colonies were fixed with methanol and stained with 0.5% crystal violet. The survival ratios were determined from the number of cells plated. Cells in each dosage group were plated in triplicate, and the experiments were repeated twice.

### Isolation of total RNA and real-time RT-PCR

We isolated total RNA from cells using TRIzol® reagent (Invitrogen) according to the manufacturer’s protocol. One microgram of total RNA was used to synthesize cDNA corresponding to the mRNA of FBP1, p21, BCCIP, TCTP, p53, and GAPDH by reverse transcription as described earlier. We used 50 ng of cDNA in Fast SYBR® Green Master Mix (Applied Biosystems), with primers directed to specific mRNA to do real-time quantitative PCR using the Fast Real PCR System (Applied Biosystems) as described [20, 21]. Data were analyzed using 7500 software (Applied Biosystems). The relative fold change in specific mRNA copies was calculated by normalizing the amount of GADPH mRNA in each sample. All experiments were done in triplicate for each data point.

### Luciferase reporter gene assay under radiation-induced stress

The reporter plasmid, p53-Luc, and positive control plasmid pFC-p53 were purchased from Agilent Technologies (Santa Clara, CA). MG-Luc plasmid (kindly provided by Dr. Bert Vogelstein) carrying a mutation in the p53 binding sites was used as a negative control [22]. WWP-Luc (also provided by Dr. Bert Vogelstein), is a reporter gene with a 2.4-kb p21WAF1 promoter region [23]. Huh7 cells were transfected with one μg of reporter vectors and 50 ng of pRL-SV40 expressing Renilla luciferase as internal control with DMRIE-C reagent (Invitrogen) according to the manufacturer’s instructions. After 48 h of transfection, luciferase activity was measured according to the manufacturer’s protocol (Promega, Madison, WI). Assays were done in four parallel sets.

### Immunoprecipitation

Huh7 cells (5 × 10^6^ cells per assay) were washed twice with warmed 1 x PBS and then lysed in cold buffer containing 50 mM Tris–HCl (pH 7.4), 150 mM NaCl, 1% Triton X-100, and 1x protease inhibitor cocktail (Roche Applied Science). We also treated the lysates with 60 μg of RNase A or 50 unit of the benzonase (Sigma) to avoid nonspecific RNA binding proteins that might be captured in FBP1 immunoprecipitation (FBP-IP) via RNA bridging. We incubated the cell lysates with 2 μg of anti-FBP1 antibody (FBP1 C-20; Santa Cruz Biotechnology) for 1 h at 4°C, then added 20 μl of protein A/G Plus agarose beads (Santa Cruz Biotechnology). The mixture was then incubated overnight at 4°C. The immunoprecipitates were collected by centrifugation at 2,500 rpm for 5 min at 4°C. After washing the pellets four times with lysis buffer, we resuspended the immunoprecipitates in 1 × Laemmli gel loading buffer. Samples were boiled and centrifuged to pellet the agarose beads. The supernatant was subjected to SDS-PAGE and Western blotted for the target proteins.

## Results

### Increased expression of p21 in FBP1 knockdown cells

We have previously shown that down-regulation of FBP1 by siRNA drastically reduced the replication of HCV subgenomic replicons in MH14 cells, whereas overexpression of FBP1 significantly enhanced virus replication [24]. FBP1 has also been shown to suppress p21 [19] cell cycle regulator. Since p21expression is also under the control of p53, it was important to examine whether knockdown of FBP1 expression influences the expression of p21 and p53 in Huh 7 cells. We, therefore, generated stable FBP-kd Huh7 cell lines NP-4 and NP-5 using FBP1 shRNA, and then analyzed them for FBP1 expression (Figure 1A, lanes 4, 5). We also used control lentivirus to generate stable cell lines NP-2 and NP-3, as controls without affecting the expression of FBP1 (Figure 1A, lanes 2, 3). Although we observed knock down of FBP1 expression by shRNA in several colonies (Figure 1A), we used the NP-4 (lane 4) colony as the stable FBP-kd cell line and the NP-2 (lane 2) colony as the lentivirus control cell line. We then examined the level of expression of p21 and p53 in control Huh7, lentivirus control (NP-2) and FBP-kd (NP-4) cells (Figure 1B, lanes 1 through 3), noting that the p53 level in both FBP-kd Huh 7 cells (NP-4) and control cells remained unchanged. In contrast, p21 expression was enhanced in FBP1 knockdown cells (lane 2) but remained suppressed in the control cells (lanes 1 and 3).

### Transient expression of FBP1 in FBP1-knockdown cells restored suppression of p21 expression

Since expression of p21 in FBP1-kd cells is significantly enhanced while remained suppressed in the presence of FBP1 in control Huh7 cells, we examined whether transient expression of FBP1 in FBP-kd cells could restore the suppression of p21 expression. We, therefore, constructed a shRNA resistant expression clone of FBP1 (pCIA-CMV-FBP_SHR_) by point mutations in the shRNA targeted region in the central and C-terminal domains of FBP1 without altering the amino acid sequence (Figure 1C). We transfected FBP1-shRNA resistant FBP1 clone (pCIA-CMV-FBP_SHR_) or empty pCIA-CMV vector in FBP-kd Huh7 cells. After 48 h of transfection, cells were γ-irradiated (3Gy) and 6 h later cells were lysed and Western blotted for FBP1, p53, p21 and actin (Figure 1D). We found that transient expression of FBP1 in FBP-kd Huh7 cells significantly suppressed p21 expression (lane 5) as compared to control FBP-kd cells (lane 3) or FBP-kd cells transfected with empty vector pCIA-CMV alone (lane 4). This also indicated that irradiated FBP-kd cells expressing transient FBP1 display p21-expression profile similar to that seen in control Huh7 cells (lanes 1 and 2).

### Knockdown of p53 or BCCIP abrogate p21 expression in Huh7 cells

p21 is positively regulated by p53. A p53 regulatory protein, BCCIP, has also been shown to regulate p21 positively via its interaction with p53. We, therefore, hypothesized that enhanced expression of p21 in FBP-kd Huh7 cells could be a result of its positive regulation by p53 and BCCIP, which may be suppressed in the presence of FBP1 in control Huh7 cells. We tested this hypothesis using Huh7 cells stably knockdown for p53 or BCCIP, and then Western blotted their cell lysates for p21 expression. We also used a colon cancer cell line HCT116 with p53 negative or positive phenotype. We found that as compared to controls, down-regulation of p53 or BCCIP in Huh7 cells drastically reduced the expression of p21 (Figure 1E, lanes 3, 4). Similar reduction in p21 expression was also observed in p53-/- HCT116 cells as compared to p53+/+ control (Figure 1E, lanes 6, 7). We found that the basal level of expression of p21 seen in control Huh7 cells (Figure 1E, lanes 1, 2) was significantly enhanced in FBP-kd cells (lane 5). These results indicate that increased expression of p21 in FBP-kd cells may be the result of activation of p53 and BCCIP, which may have remained suppressed in the presence of FBP1 in control Huh7 cells. These results further suggest that in Huh7 cells mutant p53 (Y220C) reported to be transcriptionally inactive [25], may indeed be active but its transcription activity may be strongly suppressed by FBP1.

### Expression of p53, p21, BCCIP and TCTP in control and FBP-kd Huh 7 cells in response to cellular stress

**Figure 2:**
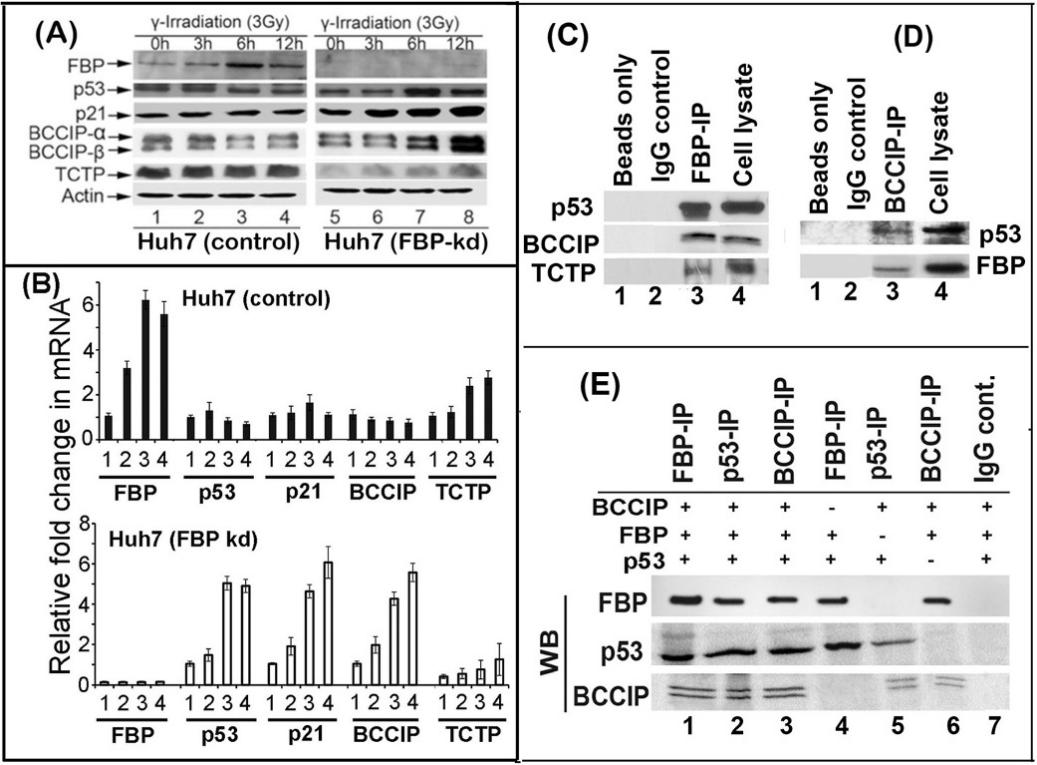
**FBP interacts with p53 and its regulatory proteins, and regulates their expression. (A)** Effect of radiation-induced stress on the expression of p53, p21, BCCIP and TCTP in control and FBP-kd Huh 7 cells. Cells grown for 48 h were irradiated with 3-Gy of γ-ray. The irradiated cells were grown for 0, 3, 6, and 12 h and examined for the expression of p53, p21, BCCIP and TCTP by Western blotting. Lanes 1-4, show results from cells grown for 0, 3, 6 and 12 h post-irradiation **(B)** Fold change in the mRNA level of FBP1, p53, p21, BCCIP and TCTP in irradiated control and FBP-kd Huh7 cells. Cells grown for 48 h were irradiated with 3-Gy of γ-ray and grown for 0, 3, 6, and 12 h. Fold changes in mRNA level of FBP1, p53, p21, BCCIP, and TCTP were determined by real-time quantitative PCR. Upper and lower panels show fold change in mRNA level in irradiated control and FBP-kd cells, respectively. Lanes 1-4, show results from cells grown for 0, 3, 6 and 12 h post irradiation. **(C, D)** Co-immunoprecipitation of FBP1, p53 and BBCIP. IP was done with either **(C)** anti-FBP1 antibody or **(D)** anti-BCCIP antibody, and the IP complexes were captured on protein A/G PLUS-agarose beads, resolved on 8% polyacrylamide SDS-PAGE gel and Western blotted for p53, BCCIP and TCTP and FBP1. **(E)** FBP, p53 and BCCIP form ternary complex in-vitro. We incubated 500 ng of each of the recombinant FBP, p53 and BCCIP at 4^o^C for 30 min. The complex was then subjected to FBP-IP, p53-IP or BCCIP-IP. The IP complex was resolved by SDS-PAGE and Western blotted for FBP, p53 and BCCIP. Lanes 1-3, show ternary complex of FBP1-p53-BCCIP captured by immunoprecipitation of FBP, p53 and BCCIP. Lane 4-6 show the formation of binary complexes between FBP1-p53, p53-BCCIP and FBP1-BCCIP, respectively.

Since p53 is involved in regulating and controlling the induction of apoptotic and growth-arrest factors in response to cellular stress, we examined whether FBP1 interferes in the function of p53 in Huh7 cells under radiation-induced stress. We irradiated control Huh 7 cells and FBP-kd cells with 3-Gy of γ-irradiation. The irradiated cells were further grown for 0, 3, 6 and 12 h, then processed for preparation of cell lysates. An aliquot of cell lysates of each set containing equivalent protein was subjected to SDS-PAGE and Western blotting for FBP1, p53, p21, BCCIP and TCTP expression. Their mRNA levels were also determined by real-time RT-PCR.We found that in control Huh7 cells, FBP1 expression was significantly increased at 6 h post-irradiation, but there was no change in the level of expression of p53 and p21 (Figure 2A, lane 3). In contrast, in FBP-kd cells, expressions of both p53 and p21 were significantly enhanced at 6–12 h post-irradiation (Figure 2A, lanes 7 and 8). These results indicate that in the presence of FBP1 in control Huh7 cells, the expression of p53 and p21 remained insensitive to irradiation treatment suggesting that the p53-mediated response to cellular stress is inhibited by FBP1. In contrast, increased expression of p21 in response to irradiation in FBP-kd Huh7 cells suggests that the transcription activity of p53 in control Huh7 cells may have been inhibited in the presence of FBP1.

We further observed upregulation of translationally controlled tumor protein (TCTP) in control Huh7 cells at all-time points (Figure 2A, lanes 1–4) while its expression level in FBP-kd Huh7 cells was drastically reduced (Figure 2A, lanes 5–8), suggesting that FBP1 may be a positive regulator of TCTP, which is also known as fortilin. TCTP is a negative regulator of p53 transcription activity, functioning as an anti-apoptotic factor by interacting with and destabilizing p53 [8, 26]. The expression level of BCCIP was marginally reduced in control Huh 7 cells after 6–12 h post-irradiation (lanes 3 and 4), but was significantly induced in FBP-kd cells within 6–12 h post-irradiation (Figure 2A, lanes7, 8). BCCIP, also called TOK-1, is an important cofactor for BRCA2 in tumor suppression, functioning as a positive regulator of p53 and a modulator of CDK2 kinase activity by interacting with p21 [9, 27, 28]. The specific induction of BCCIP in FBP-kd cells suggests that BCCIP is negatively regulated by FBP1 in Huh 7 cells. These results were confirmed by determining fold change in the mRNA levels of p21, p53, BCCIP and TCTP in irradiated control and FBP-kd Huh7 cells (Figure 2B). The mRNA levels of p53, p21 and BCCIP were significantly enhanced in FBP-kd cells within 6–12 h of post-irradiation (Figure 2B, bottom panel) which remained suppressed in the control Huh 7 cells (Figure 2B, top panel). In contrast, mRNA levels of TCTP in irradiated FBP-kd cells were drastically reduced as compared to the irradiated control Huh7 cells suggesting a positive regulation of TCTP by FBP1.

### Physical interaction of FBP1 with p53 and its regulatory factors

We addressed the potential mechanisms by which FBP1 regulates p53 via interacting with p53 regulatory proteins. To examine whether p53, BCCIP and TCTP, interact with FBP1, we carried out immunoprecipitation from the benzonase treated cell lysates of Huh 7 cells using FBP1 antibody, and then Western blotted for p53, BCCIP, and TCTP. We found that endogenous FBP1 interacts with p53, as well as its two regulatory proteins, BCCIP and TCTP (Figure 2C, and lane 3). We also did BCCIP-IP and Western blotted for FBP1 and p53. As shown in Figure 2D, BCCIP interacts with both p53 and FBP1 (lane 3). Based on these results, it can be hypothesized that binding of FBP1 with both p53 and its positive regulator BCCIP may prevent their interaction, resulting in loss of BCCIP-mediated activation of p53-transcription activity. In contrast, FBP1-mediated upregulation of TCTP expression, as well as interaction of FBP1 -TCTP complex with p53, may increase suppression of transcription activity of p53.In order to further confirm physical interaction of FBP1 with p53 and BCCIP, we also carried out immunoprecipitation on purified recombinant proteins of FBP1, p53 and BCCIP. Five hundred nanograms of each of the purified proteins were mixed together and subjected to immunoprecipitation using antibody against FBP1, p53 or BCCIP (Figure 2E). We found that FBP-IP, p53-IP or BCCIP-IP captures all three proteins indicating the formation FBP1-p53-BCCIP ternary complex (Figure 2E, lanes 1–3). We also confirmed the formation of binary complexes of FBP1 with p53 or BCCIP in addition to binary complex formation between p53 and BCCIP (lanes 4–6). These results support our hypothesis that FBP1 directly interacts with p53 and BCCIP which may be involved in suppressing p21 expression.

### Inhibition of p53 transcription activity is reversed in FBP-kd Huh7 cells

**Figure 3:**
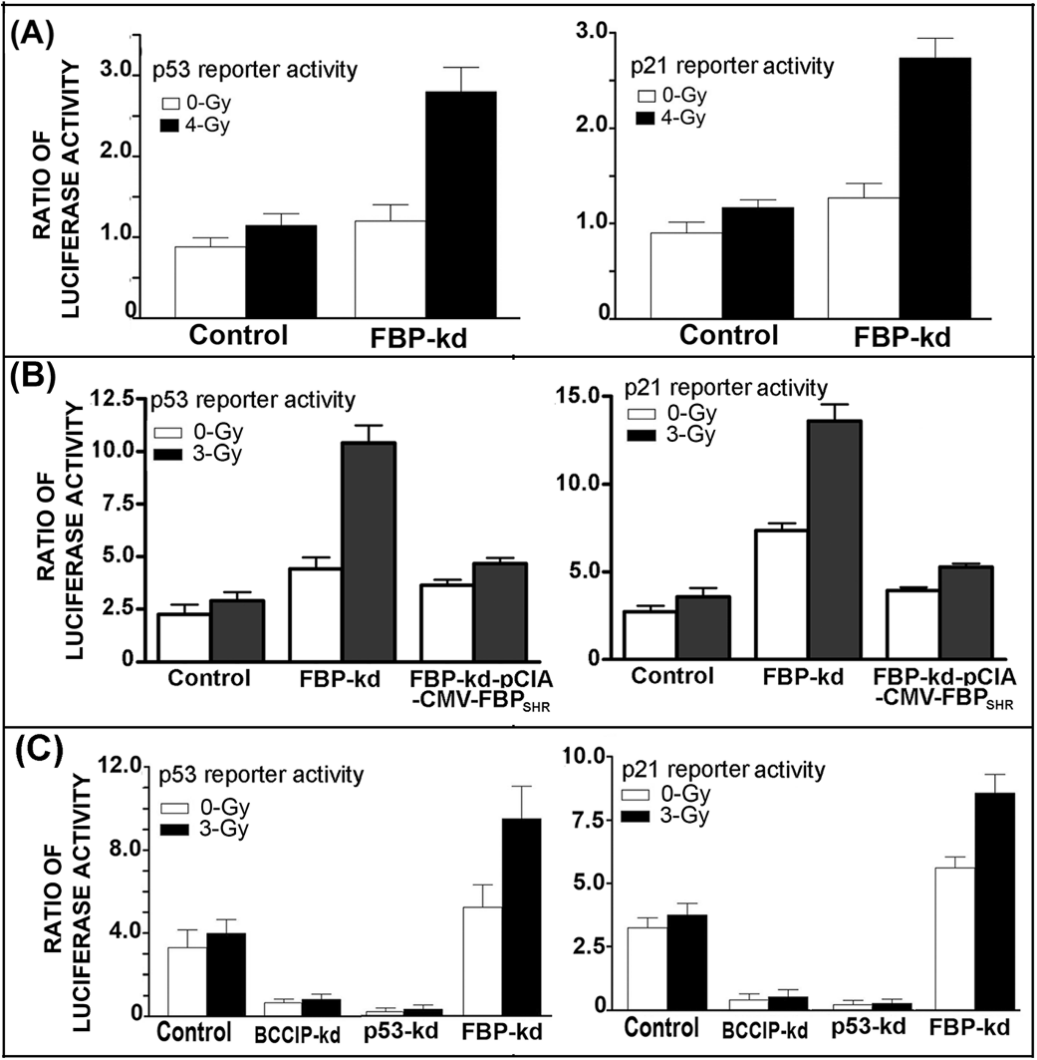
**The transactivation activity of p53 is activated in FBP-kd Huh7 cells. (A)** Activation of p53 transcription activity in FBP-kd Huh7 cells. Reporter vectors were transfected to control Huh 7 cells or FBP-kd cells as described in the Methods. Forty-eight hours later, cells were irradiated with 4 Gy of γ-irradiation. Luciferase activities were measured 6-hour post-irradiation treatment. p53 reporter activity (left panel), p21 reporter activity (right panel). **(B)** Transient expression of FBP1 in FBP-kd cells suppresses p53 transcription activities. FBP-kd cells were transfected with shRNA resistant FBP1 expression clone (pCIA-CMV-FBP_SHR_) along with reporter vectors; 48-h later cells were irradiated with 3 Gy of γ-irradiation. Luciferase activities were measured 6-hour post-irradiation treatment. p53 reporter activity (left panel), p21 reporter activity (right panel). **(C)** Relative p53 transcription activity in BCCIP-kd, p53-kd and FBP-kd cells. Huh7 cells knockdown for BCCIP; p53 and FBP1 were transfected with reporter vectors; 48-h later cells were irradiated with 3 Gy of γ-irradiation. Luciferase activities were measured 6-hour post-irradiation treatment. p53 reporter activity (left panel), p21 reporter activity (right panel).

We know that p21 is a direct target protein of p53 and that its expression is regulated by p53. We hypothesized that the transcription activity of p53 is impaired in FBP1-expressing Huh7 cells. We first used p53-Luc reporter plasmid to test this hypothesis. P53 reporter activities were increased 2.5 fold by γ-irradiation at 4-Gy dose in FBP-kd Huh7 cells while only a marginal increase of approximately 0.2 fold occurred in irradiated control cells that expressed FBP1 (Figure 3A, left panel). To confirm this, p21-Luc, which is regulated by the promoter DNA sequence of p21 gene, was transfected into control and FBP-kd Huh7 cells. As shown in Figure 3A (right panel), p21 promoter activity was enhanced by greater than 2.5 fold in FBP-kd Huh7 cells as compared to 0.25 fold increase in the control cells. These results demonstrated that p53 transcription activity in Huh7 cells is strongly inhibited by FBP1.In order to confirm that observed activation of p53 in FBP-kd cells is due to absence of FBP1, we transiently expressed shRNA resistant FBP1 in FBP-kd cells. The FBP-kd Huh7 cells were transfected with FBP1 expressing clone along with reporter plasmids. After 48 h post-transfection cells were γ-irradiated and 6 h later analyzed for expression of luciferase activity. We found that the enhanced activation of p53 reporter activity in FBP-kd cells was reversed and similar to control Huh7 when FBP1 was transiently expressed in these cells (Figure 3B, left panel). Similar results were obtained with p21 reporter activity in the absence and presence of FBP1 in FBP-kd cells (Figure 3B, right panel).

### Knockdown of BCCIP and p53 abolishes radiation-induced activation of p53 transcription activity in Huh7 cells

Since FBP1 also interacts with BCCIP, it is possible that the effect of FBP1 in suppressing p53 activity could occur through its interaction with BCCIP. Downregulation of BCCIP has been reported in many cancers [29–31]. To examine this possibility, we used stable BCCIP-knockdown or p53-knockdown Huh7 cells and used them to examine p53 transcription activity under radiation-induced stress by means of p53-Luc and p21-Luc reporters system. We found that both p53-Luc and p21-Luc reporter activities in irradiated FBP-kd cells were 2.5-3 folds higher than in the irradiated control cells while it was negligible in Huh 7 cells knockdown for either p53 or BCCIP (Figure 3C). These results clearly indicate that the FBP1-mediated inhibition of p53 transcription activity may occur as a consequence of its ability to interact with both p53 and BCCIP, and to prevent their interaction, which is essential for activation of p53 activity.

### Down-regulation of FBP1 in Huh7 cells confers radiation sensitivity

**Figure 4:**
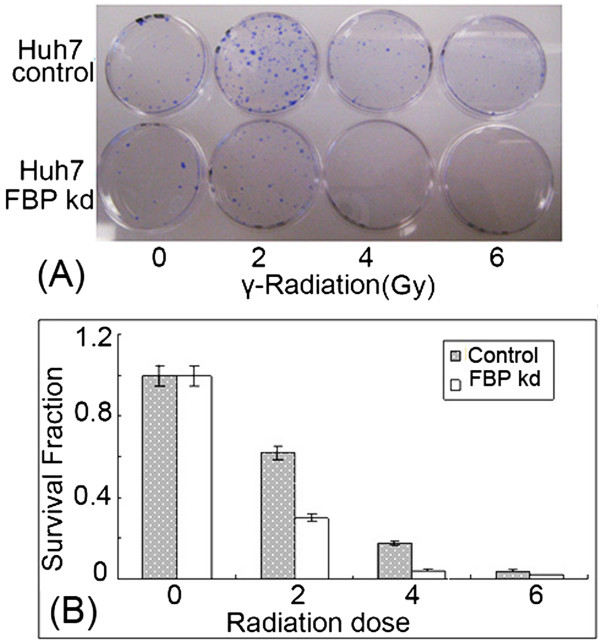
**FBP-kd increases the sensitivity of Huh7 cells to radiation damage. A**: Control and FBP –kd cells were exposed to γ-irradiation, and planted on plates. Colonies formed after two weeks of post-irradiation. **B**: The survival curves of control and FBP -kd cells exposed to radiation treatment.

p53 is a key protein in the cell cycle and programs cell death by regulating the expression of several cellular factors. Lack of the transcription activity of p53 is always associated with cell resistance to radiation. Using reporter vectors, we found that FBP1 down-regulation in Huh7 cells activated p53 transcription activities. To confirm whether FBP1 is associated with radiation resistance by inhibiting p53 transcription activity in Huh7 cells, both control Huh7 and FBP-kd Huh7-cells were exposed to irradiation, and colony formation was measured. As shown in Figure 4A,B, FBP-kd cells showed higher sensitivity to radiation treatment than did control Huh7 cells, strongly suggesting that FBP1 is important in impairing p53 transcription activity in Huh7 cells.

Based on these results, we conclude that FBP1 physically interacts with p53 and suppresses its transcription activity by (i) directly inhibiting p53, (ii) by upregulating TCTP and (iii) by down-regulating BCCIP. FBP1 not only interacts with and down-regulates BCCIP, which is required for activation of p53 activity, but also interacts with and upregulates TCTP that negatively regulates the stability of p53 and thus negatively affect p21 expression.

## Discussion

FBP1 is a transactivator of the *c-myc* gene, which is abundantly expressed in many cancers including HCC tumors [19, 32, 33]. Earlier we reported that FBP1 is essential for HCV replication in MH14 cells derived from Huh7 cells, an hepatocellular carcinoma cell line in which FBP1 is abundantly expressed [24]. Knockdown of FBP1 in these cells drastically reduced HCV replication [24]. In another study, Dharel et al. [34] have reported that in Huh7 cells, p53 exerts an inhibitory effect on HCV replication while knockdown of p53 makes these cells more permissive of viral replication. In Huh7 cells p53 contains an Y220C mutation that is located outside of the conserved p53-DNA binding domain and therefore not essential for the tertiary structure of the DNA binding surface [25, 35]. Kubicka et al. [25] have shown that in Huh7 cells, the mutant p53 (Y220C) is not only dominant negative but lacks p53-dependent target gene activation activity. It has been shown that most cancer mutation are located on DNA-binding core domain of p53 consisting of central β-sandwich [36] that has been shown to be temperature sensitive and had destabilizing effect on p53 at physiological temperature [37]. Unexpectedly, Y220C mutation located 38 Å away from the DNA binding core on the periphery of the β-sandwich, has also been shown to destabilize p53 and affect its function at physiological temperature [37]. Other studies have shown that transactivation activity of Y220C mutant p53 in Huh7 cells is similar to that in Chang liver cells and HepG2 cells containing wild-type p53 [38–40]. Dharel et al. have also shown that mutant p53 in Huh7 cells are transcriptionally active and exhibits effective transactivation activity [34].

It is possible that in Huh7 cells, transactivation activity of p53 is suppressed by some specific cellular factor such as FBP1 and is not entirely inactivated due to Y220C mutation. It is well known that binding of SV-40 large-T-antigen to the wild-type p53 completely inactivates its transcription activity [41]. In this context, we found that FBP1 strongly and independently interacts with p53 as well as with the p53-regulatory proteins, BCCIP and TCTP, and therefore may be responsible for the suppression of p53 activity in Huh7 cells. This possibility was supported by our finding that upon radiation-induced stress, expression of both p53 and p21 was significantly enhanced in FBP-kd cells but remained nearly unchanged in control Huh7 cells (Figure 2A,B). We further found that FBP1 modulates the expression of TCTP and BCCIP, which, respectively, function as negative and positive regulators of p53. Under radiation-induced stress, the expression of TCTP is strongly decreased in FBP-kd cells while BCCIP expression is significantly induced within 6–12 h after irradiation (Figure 2A,B). Increased BCCIP expression under conditions of cellular stress may enhance p53 transactivation activity. Similarly, down-regulation of TCTP expression may relieve p53 from its inhibitory effect in FBP1 knockdown Huh7 cells. Using reporter assay, we confirmed significant transactivation of p53 activity in FBP-kd cells but nearly inactive in control Huh7 cells (Figure 3A) and completely abolished in BCCIP-kd or p53-kd cells (Figure 3C). In order to confirm that observed activation of p53 activity in FBP-kd cells is due to positive regulation of p53 in the absence of FBP1, we carried out transient expression of shRNA resistant FBP1 in FBP-kd cells. We found that the transient expression of FBP1 strongly inhibited activation of p53 activity similar to as observed in the control Huh7 cells (Figure 3B).

The p53 has two major functions including negative regulation of cell growth and the induction of apoptosis [42]. Following exposure to ionizing radiation, cells with functional p53 are arrested in G_1_ phase while cells with non-functional p53 are not arrested at G1 checkpoint [42]. Huh7 cells are significantly resistant to radiation-induced stress [43] that may largely be attributed to the presence of inactive p53 [44]. We also found significantly high resistance in Huh7 cells exposed to γ-irradiation. In contrast, FBP-kd Huh7 cell was highly sensitized to γ-irradiation as compared to the control Huh7 cell. At the exposure of as low as 2Gy, approximately 70 % of the loss of cell survival in FBP-kd cells occurred, while only 22 % of control Huh7 cells failed to survive (Figure 4B) suggesting that FBP1 suppressed the p53-mediated cellular response to radiation-induced stress. High expression of FBP1 in Huh7 cells inhibits p53-mediated response to irradiation treatment, while FBP-kd restores the sensitivity of Huh7 cells to irradiation. Thus, FBP1 has a critical function in regulating p53 function, a key DNA intact keeper and tumor suppressor. Recently, Liu et al. have shown that binding of small molecule, PK7088, to Y220C mutant p53 in Huh7 cells restored its transcription activity similar to that of wild-type p53 [45]. It would be interesting to examine whether PK7088 disrupts the interaction of FBP1 with either mutant p53 or with BCCIP in Huh 7 cells which may result in abrogating FBP1 mediated inhibition of p53 function.

FBP1 was first found as a single-strand binding protein that destabilizes the nucleic acid duplex [46, 47]. FBP1 binds the pyrimidine-rich FUSE of human *c-myc* proto-oncogene and activates *c-myc* transcription [18, 48]. In a mouse xenograft transplantation model, knockdown of FBP1 in hepatocellular carcinoma cells resulted in high sensitivity to cell death treatment, lowered proliferation, and tumor formation [19, 49]. It has recently been shown that JTV1/AIMP2/p38, a structural subunit of a multi-amino acyl-tRNA synthetase (ARS) complex, interacts with FBP1 and induces the expression of ubiquitin-specific peptidase 29 (USP29) which, in turn, stabilizes p53 by promoting deubiquitination [50]. It may be possible that FBP1 may have an inhibitory effect on USP29 expression that may be relieved by JTV1/AIMP2/p38 mediated sequestering of FBP1. This contention is supported by the fact that FBP1 is downregulated by JTV1/AIMP2/p38 [51]. In contrast, a splicing variant of AIMP2/p38 identified as DX2 competes with AIMP2/p38 for binding to p53 and promotes MDM2-mediated degradation of p53 [52]. It will be interesting to examine whether DX2 also competes with AIMP2/p38 for binding to FBP1 and inhibits FBP1 mediated induction of USP29, which in turn, may have destabilizing effect on p53.

In this study, we demonstrated that FBP1 inhibits cell sensitivity to irradiation, one means of treatment to obtain cell death; It also impairs p21 expression by suppressing p53 transactivation activity. The tumor harboring p53 mutation is often defective in transcription activity [53]; mice expressing defective p53 transcription activity are predisposed to tumors [4]. In many of the tumors, the tumor-suppressing activity of p53 may be subverted or functionally compromised by other mechanisms. Many cellular factors such as antagonism by TCTP [8], MDM2, Sir2α [54] or by factors derived from pathogens such as Tax from human T-cell leukemia virus type 1 [55], SV40 T large antigen [41], adenovirus E1B 55 K protein [56], and hepatitis B virus X protein [57] are involved suppressing the p53 activities.

By interacting with p53, TCTP blocks p53-induced transcription activation of Bax and inhibits p53-dependent apoptosis. Abrogation of the interaction between p53 and TCTP-like protein can retrieve p53 tumor-inducing activity. Small molecules, such as nutlins and RITA can retrieve p53 tumor-suppressing activity by inhibiting the interaction between MDM2 and p53 or binding with p53. Our work clearly establishes FBP1 as a potential target, just like MDM2, in the effort to develop drug that are active against p53-bearing tumors. P53 transcription activities refer to several factors, including Serine phosphorylation [58], C-terminal acetylation [59, 60] and tetramer formation [61–63] and which of these mechanisms is disrupted by interaction with FBP1 is yet to be established.

## Conclusion

It is our novel discovery that FBP1, which is abundantly expressed in HCC tumors, is involved in the suppression of transactivation activity of p53 by physically interacting with p53 as well as its two regulatory proteins BCCIP and TCTP. The p53 transcription activity is enhanced in FBP-kd cells resulting in increased sensitivity to radiation. Since FBP1 expression in normal differentiated liver cells is very low, our results suggest that over expression of FBP1 may be involved in promoting HCC tumor by suppressing p53 activity and could be a potential target for drug development.

## Abbreviations

FUSE: Far upstream element
FBP1: FUSE binding protein1
BRCA2: Breast cancer type 2 susceptibility protein
CDKN1A: Cyclin-dependent kinase inhibitor 1A (p21/WAF1)
BCCIP: brca2 and cdkn1a interacting protein
TCTP: Translationally controlled tumor protein
MDM2: Murine double minute 2 homolog
PUMA: p53 upregulated modulator of apoptosis
MCL1: Myeloid cell leukemia sequence 1.
